# The feasibility of using exoskeletal‐assisted walking with epidural stimulation: a case report study

**DOI:** 10.1002/acn3.50983

**Published:** 2020-02-05

**Authors:** Ashraf S. Gorgey, Satinder Gill, Matthew E. Holman, John C. Davis, Roozbeh Atri, Ou Bai, Lance Goetz, Denise L. Lester, Robert Trainer, Timothy D. Lavis

**Affiliations:** ^1^ Spinal Cord Injury and Disorders Center Hunter Holmes McGuire VAMC 1201 Broad Rock Boulevard Richmond Virginia 23249; ^2^ Department of Physical Medicine & Rehabilitation Virginia Commonwealth University Richmond Virginia 23298; ^3^ Department of Electrical and Computer Engineering Florida International University Miami Florida 33174; ^4^ Physical Medicine & Rehabilitation Hunter Holmes McGuire VAMC 1201 Broad Rock Boulevard Richmond Virginia 23249

## Abstract

Spinal cord epidural stimulation (SCES) exhibits a rehabilitation potential of restoring locomotion in individuals with spinal cord injury (SCI). However, this is linked to an intensive rehabilitation locomotion approach, which is impractical to apply among a large clinical SCI population. We, hereby, propose a rehabilitation approach of using SCES to enhance motor control during exoskeletal‐assisted walking (EAW). After 24 sessions (12 weeks) of EAW swing assistance decreased from 100% to 35% in a person with C7 complete SCI. This was accompanied by 573 unassisted steps (50% of the total number of steps). Electromyographic pattern improved during EAW, reflecting the subject’s ability to rhythmically activate paralyzed muscles. Rate perceived exertion increased during EAW with SCES compared to stepping without SCES. These preliminary findings suggest that using SCES with EAW may be a feasible rehabilitation approach for persons with SCI.

Survivors with clinically complete SCI are confined to a wheelchair for mobility.^1^ The transition from free, over ground ambulation to wheelchair use results in adverse changes in body composition, cardiovascular health, and a significant socioeconomic burden.^2^, ^3^, ^4^, ^5^ Restoration of locomotion has been the focus of years of research aimed at ameliorating several comorbidities and securing independence after SCI.^2^, ^6^ Despite these efforts, few have offered potential solutions for the restoration of locomotion after SCI, partially because of the high metabolic demand and a reliance on upper extremity muscles during walking.^2^, ^6^

Spinal cord epidural stimulation has emerged as a tool to activate and control lower extremity muscles to stand, step, and walk, both with and without assistive devices.^7^ SCES of the lumbar segments has restored mobility in the paralyzed muscles of rats and other nonhuman primates.^8^, ^9^, ^10^, ^11^, ^12^ Recent reports have demonstrated that SCES can serve as a neuromodulation technique to enhance rhythmic and tonic motor patterns in persons with complete SCI.^13^, ^14^, ^15^ In order to ensure sensorimotor reeducation of the spinal circuitry and central pattern generators consistent with walking, SCES must be accompanied with intensive locomotor training.^7^, ^16^ A recent report indicated that 43 weeks of body weight supported treadmill training were needed to improve overground walking speed from 0.05 m·s^−1^ to 0.20 m·s^−1^ in a person with a T6 injury.^16^ Another study showed that overground walking was achieved after 85 weeks (278 sessions) of SCES in one person with a mid‐cervical SCI.^7^ These interventions require labor intensive commitments from multiple well‐trained personnel, making clinical applications of SCES difficult due to increasing number of SCI cases per year, lack of trained staff, cost involved in rehabilitation as well as commitment from the patient and their families. In our previous work, we have shown improvements to different walking parameters during EAW in persons with incomplete and complete SCI.^2^, ^17^ EAW is designed to offer overground weight‐bearing gait while allowing various levels of lower extremity movements based on the level of swing assistance provided by the device. A detailed description about different levels of swing assistance provided by EAW was published elsewhere.^2^ Therefore, it provides an opportunity to facilitate walking at a low‐metabolic cost for persons with SCI when walking with 100% swing assistance.^2^, ^18^

Additionally, despite limited walking speeds (<0.4 m·s^−1^), EAW is also less labor intensive for the rehabilitation teams^2^. High‐intensity locomotor training has been linked to improving gait speed and muscle coordination.^19^, ^20^ In this work, EAW intensity was increased by lowering the swing assistance across 24 sessions (12 weeks). Moreover, the intensity of the training remarkably increased as indicated by high rate of perceived exertion (RPE) during EAW with SCES. The primary goal of the work was to propose an alternative rehabilitation approach by determining the feasibility of using EAW with SCES to improve motor function in persons with SCI.

We present a case of a C7 clinically complete participant with SCI who had an epidural stimulator implanted over spinal segments T12‐S2 and underwent 12 weeks of EAW. Sessions 1–3 (week 1–week 2) were carried out with 100% swing assistance and no SCES. During session 4 (end of week 2), a brief period of 95% swing assistance was initiated with the intent of exposing any potential gains in locomotor control as evidenced by reduced reliance on EAW swing assistance. It was determined that additional EAW training was necessary before the swing assistance could be further reduced. At session 5 (beginning week 3), the participant was transitioned from utilizing a walker to Canadian crutches during his EAW sessions. Additionally, the participant was encouraged to briefly activate his epidural stimulator during sessions 4–6 with the goal to achieve familiarization with the stimulation.

Sessions 7–16 (week 4–8) began with 100% swing assistance that was then reduced based on the subject’s performance to walk at least 45 min during a session. By session 8 (end of week 4), the level of swing assistance was lowered from 100% to 70%, while the stimulator voltage increased from 6.0 to 8.0 V (Fig. 1A). During session 10 (end of week 5), the EAW assistance was decreased to 60% and the stimulator voltage was also lowered to 5.5–6.5 V. During session 16 (week 8), EAW assistance was decreased to 50% using only 5.0 V. By session 20 (week 10), the EAW assistance was decreased to 35% and the stimulation voltage was dropped to 4.4–4.8 V. EAW assistance was maintained at 35% for the rest of the study sessions. It should be noted that the stimulation intensity was progressively lowered by the participant over time based on his level of perceived gaiting performance and reported exhaustion. This information was paired with observations made by the research team regarding his observed performance and level of exhaustion. The number of unassisted steps (initiated and completed by participant to move his leg into extension in less than 2.5 sec) were counted during last 4 weeks of training (sessions 17–24).

**Figure 1:**
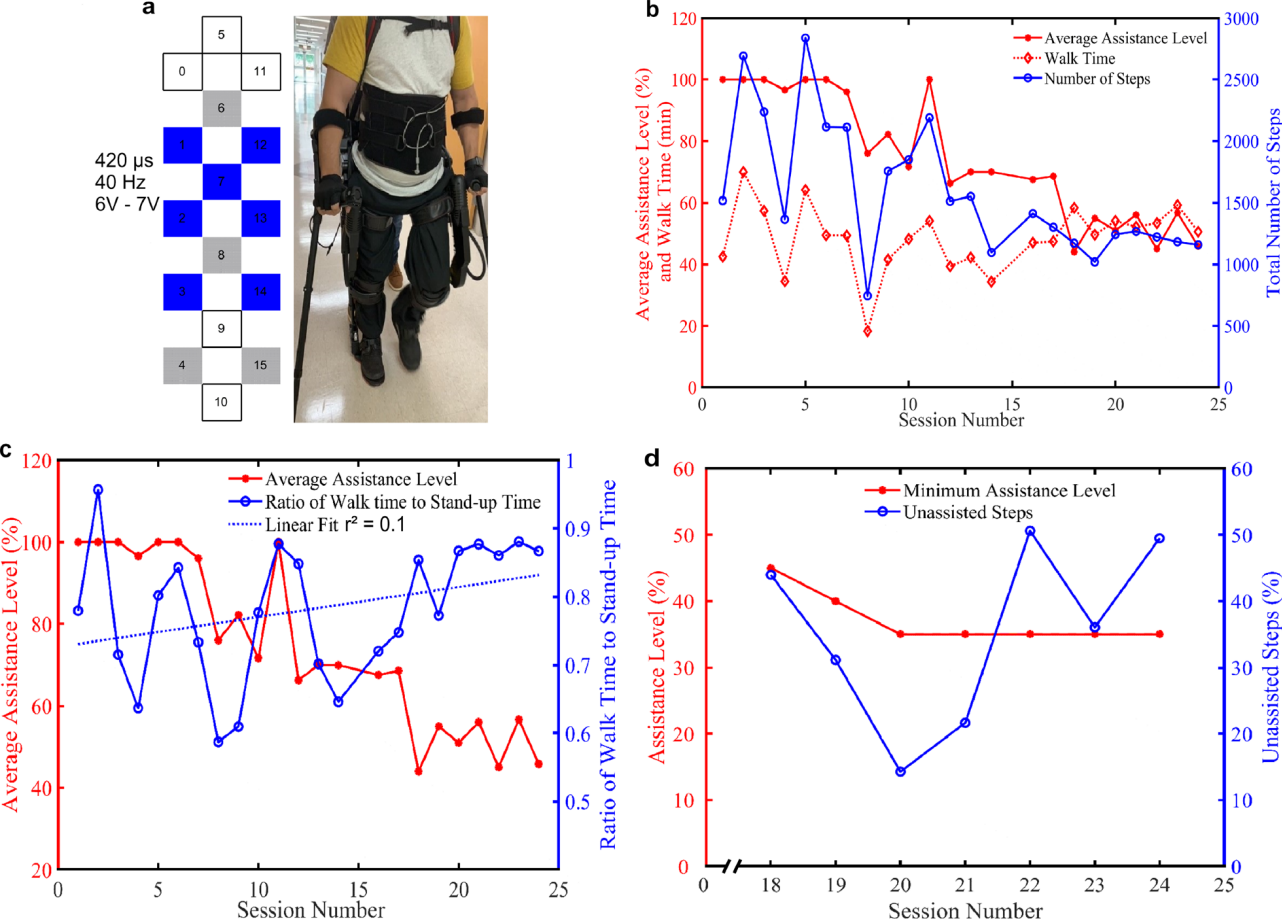
Progression of SCES enabled EAW. (A) Schematic and settings of SCES program used for stepping. Cathodes (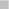); anodes (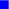); inactive (□). (B) Total number of steps taken and walk time for each session as the assistance level decreased over 12 weeks (24 sessions). During each session, multiple assistance levels were tested, results show average assistance level during each session. (C) Ratio of walk time to stand‐up time for each session as the assistance level changed over 24 session. Results showed the average assistance level during each session. (D) The minimum level of assistance and the percentage unassisted steps (percentage of the total number of steps) for sessions 18–24 are displayed.

The total number of steps taken during each session over the entire 24 sessions (12 weeks) of training are presented in Figure 1B. As the assistance level was decreased, the variability in the total steps taken stabilized along with walking time. As the assistance was decreased over 24 sessions the ratio of walk time to stand‐up time increased from 0.77 to 0.86 as depicted by the dotted blue line of best fit (Fig. 1C). This performance improvement appears to be attributed to the combined use of SCES and EAW. A collection error occurred during session 17 resulting in data loss related to the number of unassisted steps; therefore, unassisted step data are only presented starting from session 18. As the minimum assistance level was reduced from 45% to 35% between sessions 18 and 20, the percentage of unassisted steps also decreased in a linear fashion (Fig. 1D). However, when the minimum assistance level was maintained at 35% throughout sessions 20–24, the percentage of unassisted steps increased threefold (573 steps; Fig. 1D).

Surface electromyography (EMG) was captured from the quadriceps femoris (QF), hamstrings (HS), soleus (SL), and gastrocnemius (GS) of the right lower extremity at week 0 (baseline) during 100% EAW–no SCES (100‐NS) and at week 13 (post‐intervention) during 100‐NS, EAW–with SCES (100‐S), 35% EAW–no SCES (35‐NS), and 35% EAW–with SCES (35‐S). Figure 2A and B shows filtered EMG root mean square (RMS) envelopes averaged across 10 steps for QF, HS, SL, and GS during baseline (100‐NS) and post‐intervention (100‐NS and 100‐S). Baseline (100‐NS) results display rhythmic activity specifically coordinated with initial contact events. Muscle activity at post‐intervention (100‐NS) clearly demonstrates improved coordination at initial contact events when compared to baseline measures. The addition of epidural stimulation during the post‐intervention (100‐S) when compared to (100‐NS) appears to supplement the motor gains achieved through the 12‐week training period. RMS envelope correlation results (R‐values) averaged over 10 steps during post‐intervention for 100‐NS (QF: 0.36, HS: 0.34, SL: 0.21, and GS: 0.36) and 100‐S (QF: 0.61, HS: 0.55, SL: 0.31, and GS: 0.63) were generally higher when compared to baseline results for 100‐NS (QF: 0.27, HS: 0.15, SL: 0.39, and GS: 0.58). Figure2C shows filtered EMG RMS envelopes averaged across 10 steps during post‐intervention (35‐NS and 35‐S) for the QF, HS, SL, and GS. The gains observed between baseline and post‐intervention at 100‐NS, are similarly displayed during the 35‐NS condition. Moreover, during the 35‐S condition the SCES again subsidized the motor patterns when compared to 35‐NS. RMS envelope correlation results averaged over 10 steps during post‐intervention for 35‐NS (QF: 0.30, HS: 0.43, SL: 0.25, and GS: 0.28) and 35‐S (QF: 0.75, HS: 0.83, SL: 0.65, and GS: 0.62) were also generally higher than baseline results.

**Figure 2:**
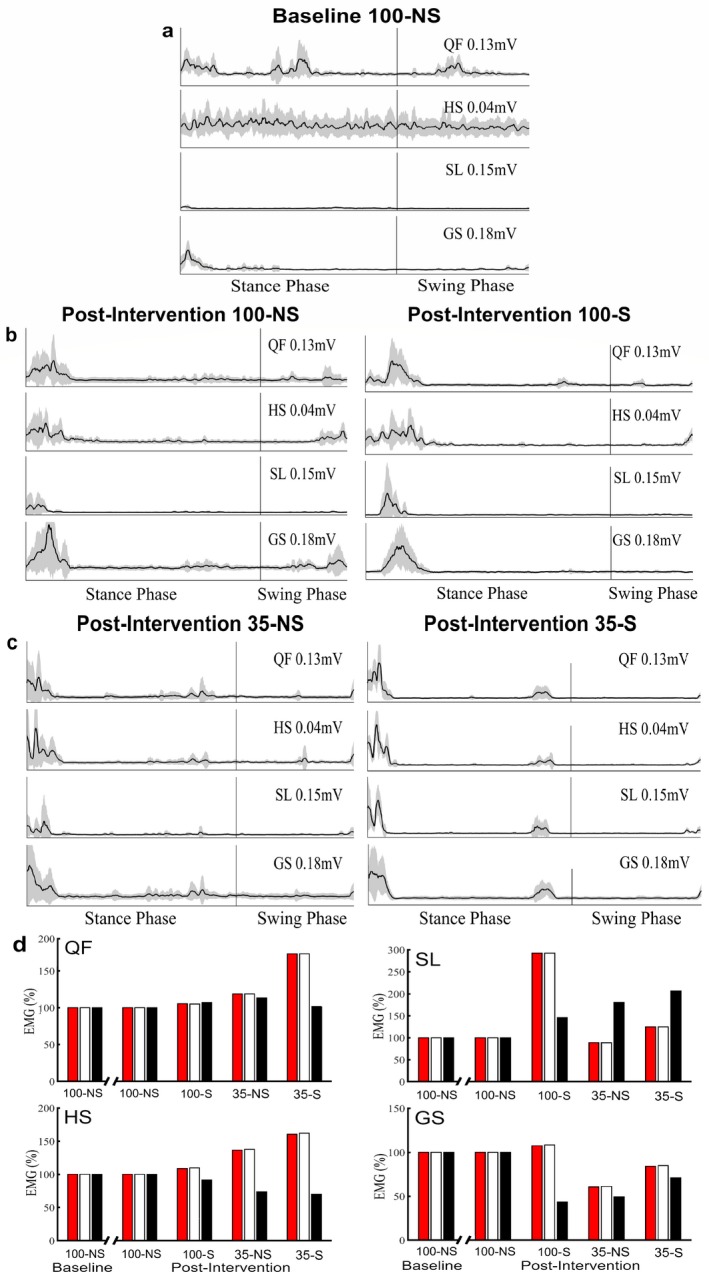
EMG activity during week 0 (baseline) and week 13 (post‐intervention) of SCES enabled EAW. (A) Filtered averaged RMS envelopes across 10 strides for QF, HS, SL, and GS of the right lower extremity during baseline (100‐NS). (B) Filtered averaged RMS envelopes across 10 strides for QF, HS, SL, and GS of the right lower extremity during post‐intervention (100‐NS and 100‐S). (C) Filtered averaged RMS envelopes across 10 strides for QF, HS, SL, and GS of the right lower extremity during post‐intervention (35‐NS and 35‐S). (D) Average filtered EMG peaks for the QF, HS, SL, and GS during various levels of assistance, across the entire stride (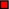), the stance phase (□), and the swing phase (■) of the right lower extremity during baseline and post‐intervention. QF, quadriceps femoris; HS, hamstrings; SL, soleus; GS, gastrocnemius; 100‐NS, 100% EAW assistance with no SCES; 35‐NS, 35% EAW assistance with no SCES; 35‐S, 35% EAW assistance with SCES. Shaded area for RMS envelopes represents the total area that falls between one standard deviation above and one standard deviation below the mean. Averaged filtered EMG peaks for the QF, HS, SL, and GS results are normalized with respect to 100‐NS during baseline and post‐intervention, respectively.

Overall following the 24 session (12‐week) training period, we observed improvement in motor patterns specifically within QF and HS muscles. This observation seems particularly enhanced through the addition of SCES during EAW and/or through lowering of the swing assistance during EAW. Figure 2D shows average filtered EMG peaks for each muscle group at various levels of assistance for both baseline and post‐intervention. Average EMG activity, particularly in QF and HS, increased as the assistance was dropped and appeared amplified by SCES. Raw filtered EMG signals over three strides for each muscle and condition displayed are provided in Figure S3.

Figure 3A and C shows filtered EMG RMS envelopes averaged across 10 steps for QF, HS, SL, and GS during baseline (100‐NS) and post‐intervention (100‐NS) for a control participant (EAW only, no SCES). The RMS envelope data for QF and HS shows improvement in a random and reflexive pattern. Raw filtered EMG signals over three strides for each muscle are provided in Figure 3B and D. RMS envelope correlation results (R‐values) averaged over 10 steps during post‐intervention for 100‐NS (QF: 0.16, HS: 0.27, SL: 0.26, and GS: 0.17) were generally smaller than baseline results for 100‐NS (QF: 0.40, HS: 0.12, SL: 0.52, and GS: 0.54).

**Figure 3:**
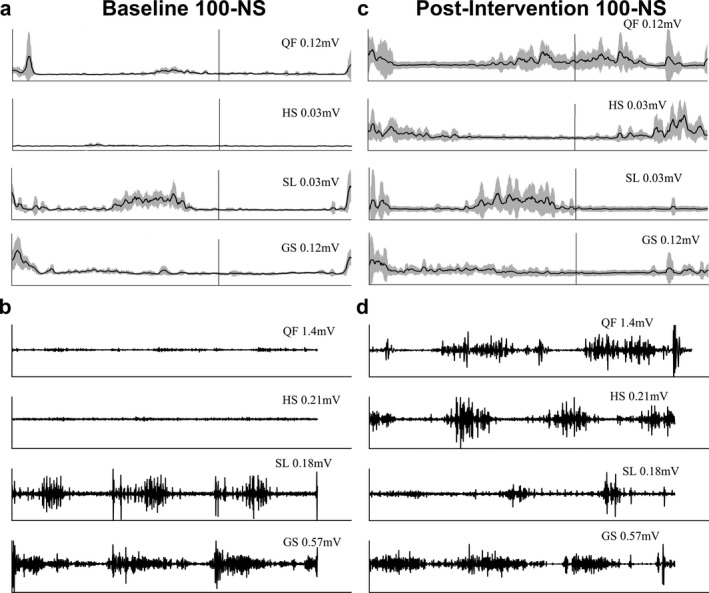
EMG data from a participant serving as a control (EAW only). (A) Filtered averaged RMS envelopes across 10 strides for QF, HS, SL, and GS of the right lower extremity during baseline (100‐NS). (B) Filtered EMG across three strides for QF, HS, SL, and GS of the right lower extremity during baseline (100‐NS). (C) Filtered averaged RMS envelopes across 10 strides for QF, HS, SL, and GS of the right lower extremity during post‐intervention (100‐NS). (D) Filtered EMG across three strides for QF, HS, SL, and GS of the right lower extremity during post‐intervention (100‐NS). QF, quadriceps femoris; HS, hamstrings; SL, soleus; GS, gastrocnemius; 100‐NS, 100% EAW assistance with no SCES. This data was acquired from a different participant. This 26‐year‐old male’s SCI was classified as AIS‐C with a C6 level of injury which occurred ~2 years prior to testing. Following 24 sessions of EAW training (no SCES), EMG activity appears repetitively unaffected within the distal musculature (SL and GS), while marked changes occurred proximally (QF and HS). QF activity during post‐intervention testing appears to be somewhat random and reflexive in nature; this is clearly evidenced by the multiple RMS peaks surrounding toe‐off and heel‐strike.

Exoskeletal‐assisted walking speed increased twofold when comparing baseline (100‐NS) to post‐intervention (100‐NS). Such improvement could have occurred because of motor learning of appropriate weight shifting strategy during EAW that was anecdotally observed; this could also facilitate cyclical limb movements (Fig. S4). However, enhanced lower extremity functional improvements were also observed as evidenced by changes in EMG patterns as presented in Figure 2. Moreover, as the assistance level dropped EAW speed decreased. The decrease in EAW assistance provided the subject with a period of 2.5 sec to allow him the opportunity to move his limb during swing phase.

Using a modified Borg scale, EAW with SCES demonstrated a dramatic increase in RPE compared to EAW without SCES. Resting RPE across all sessions was 6.1 ± 0.4 (mean ± SD) and increased to 11 ± 2.4 during 100‐NS, and to 14 ± 2 during 100‐S; then remarkably increased to 17.4 ± 1.5 and 15.7 ± 3.3 during 70‐S and 35‐S, respectively. The increases in RPE reflect an increase in the intensity of the exercise while reducing the EAW assistance level. Additionally, body composition improved from baseline to post‐intervention as demonstrated by decreased regional and total body fat mass (Table S1). Such shifts in body composition complement observed changes reported in a different study examining the combinatory effects of SCES during locomotor training among motor complete SCI participants.^21^ This demonstrated that the improvement in motor function is not due to increase in lean mass but rather to neuromodulation in the motor pattern.

Given the scope of this case report there are some limitations which future studies can address. The sample size (*n* = 1) does not allow for solid conclusions regarding the combined effects of EAW + SCES; larger and randomized clinical trials will be necessary to clarify these impacts. To provide some insight into these combinatory effects, we have provided additional EMG data collected from another individual with similar SCI demographics who underwent 12 weeks (24 sessions) of EAW only training.

Moreover, the appeal of noninvasive tools such as transcutaneous spinal stimulation (TSS) cannot be overlooked. Studies exploring the combinatory effects of EAW + TSS have demonstrated functional gains in a single participant.^18^ Future studies are warranted to confirm the beneficial effects of EAW + TSS on gaiting behaviors/outcomes; in addition, comparing both stimulation modalities may provide clinicians with multiple therapeutic tools for the rehabilitation of persons with SCI. Next, we did not have access to information regarding which variable (torque, displacement, or both) was modified when swing assistance level was reduced. This knowledge may have provided additional insights into the effects of EAW + SCES.

Finally, in the current report, stimulation amplitude was adjusted based on subjective feedback from the participant. This may have influenced the outcome of the current trial because SCES can modulate the motor pattern via different mechanisms. Therefore, testing the effects of SCES parameters on the sensory–motor system is crucial to interpret safety and motor adaptation of the current findings. This could have been accomplished by testing the effects of the same SCES parameters under different conditions (i.e., sitting, standing in the exoskeleton, walking in the exoskeleton with and without volitional involvement of the participant). Future studies need to determine whether stimulation amplitude was below, near, or above motor threshold as well as whether stimulation drove rhythmic activity, and whether descending input played a role in motor pattern generation.

In summary, 24 sessions (12 weeks) of EAW with SCES resulted in volitional stepping even after lowering the swing assistance to 35%. This was accompanied with temporal and rhythmic improvements to the EMG patterns of the lower extremity musculatures. This study also demonstrated an increase in cardiovascular demand with SCES as evidenced by increasing RPE. Finally, the participant also showed increases in EAW speed and modest improvements in body composition profile from baseline to post‐intervention.

## Conflict of Interest

The authors declare no competing interest.

## Supporting information


**Figure S1.** AIS medical examination results.

**Figure S2.** Dual‐energy x‐ray scan of the participant at week 0 showing the location of the epidural stimulator.

**Figure S3.** Filtered EMG across three strides during week 0 (baseline) and week 13 (post‐intervention) of SCES enabled EAW.

**Figure S4.** Walking speed of SCES enabled EAW. Speed during 10 m walk test for various assistance levels at baseline and post‐intervention.

**Table S1.** Body composition assessment.

## Data Availability

All data will be available from the corresponding author upon request.
