# Supplementary figures and images for: The feasibility of using exoskeletal‐assisted walking with epidural stimulation: a case report study
Source: Ann Clin Transl Neurol. 2020 Feb 5;7(2):259–65. doi: 10.1002/acn3.50983 (PMC7034511; doi:10.1002/acn3.50983)

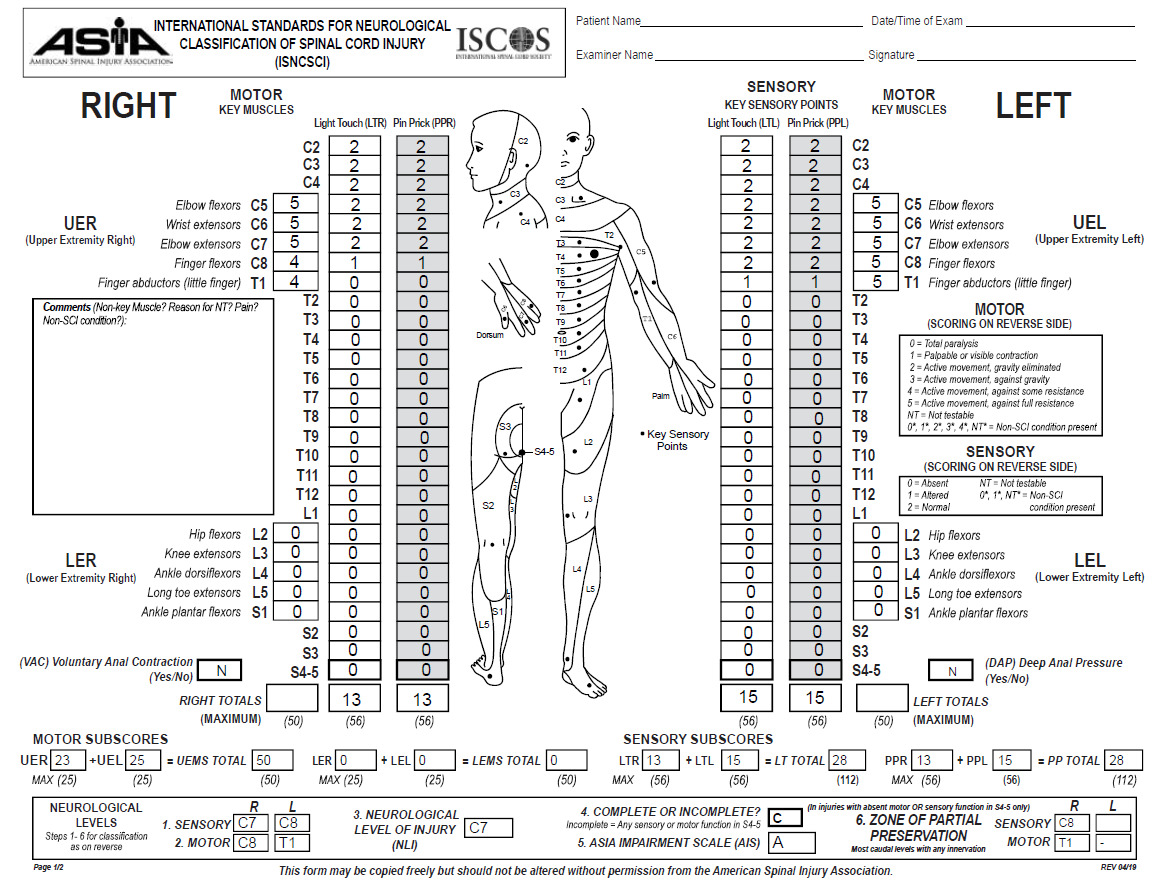

Supplement: Supplementary file 1 — Figure S1. AIS medical examination results. [file ACN3-7-259-s001.tiff]

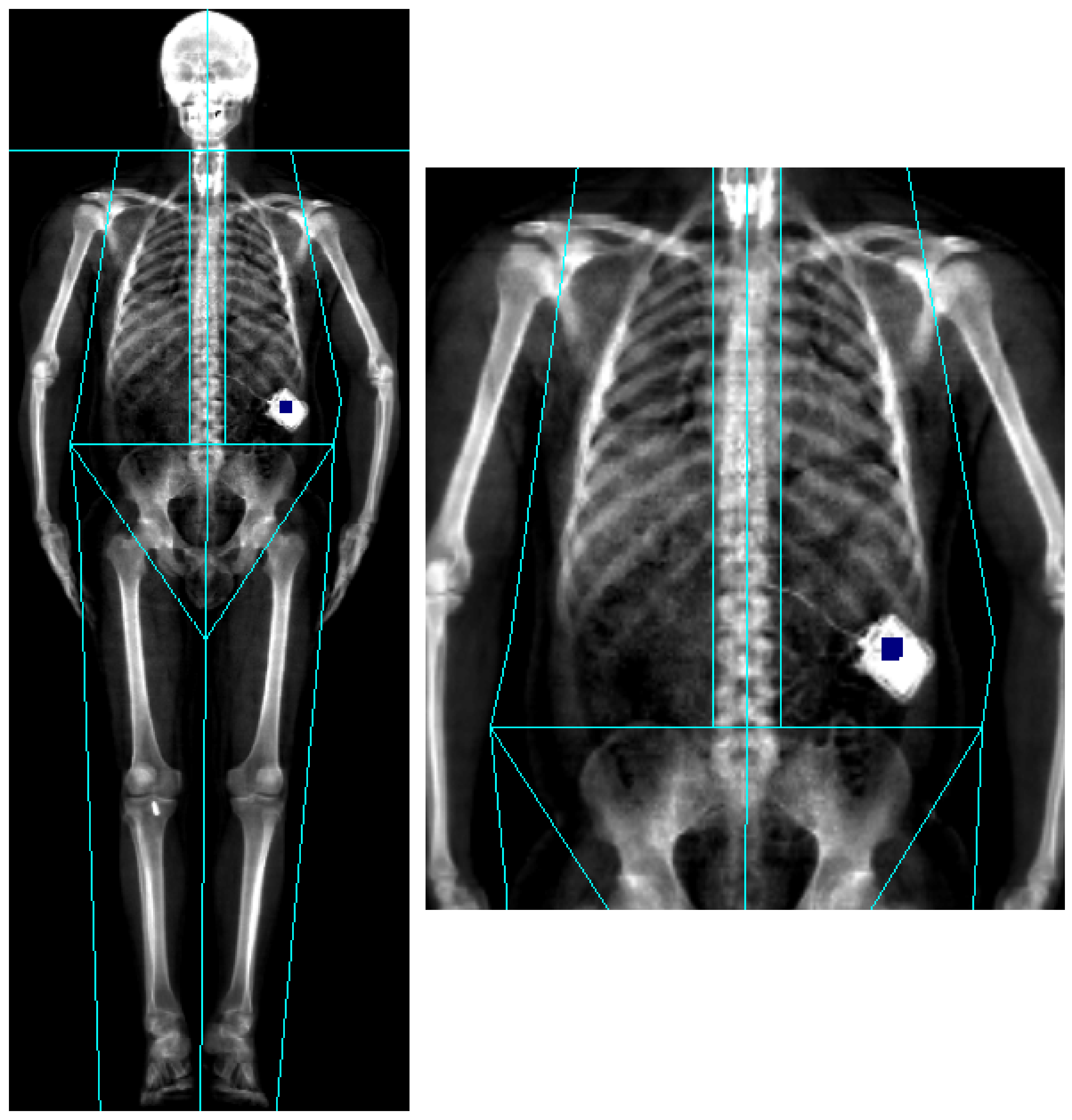

Supplement: Supplementary file 2 — Figure S2. Dual‐energy x‐ray scan of the participant at week 0 showing the location of the epidural stimulator. [file ACN3-7-259-s002.tiff]

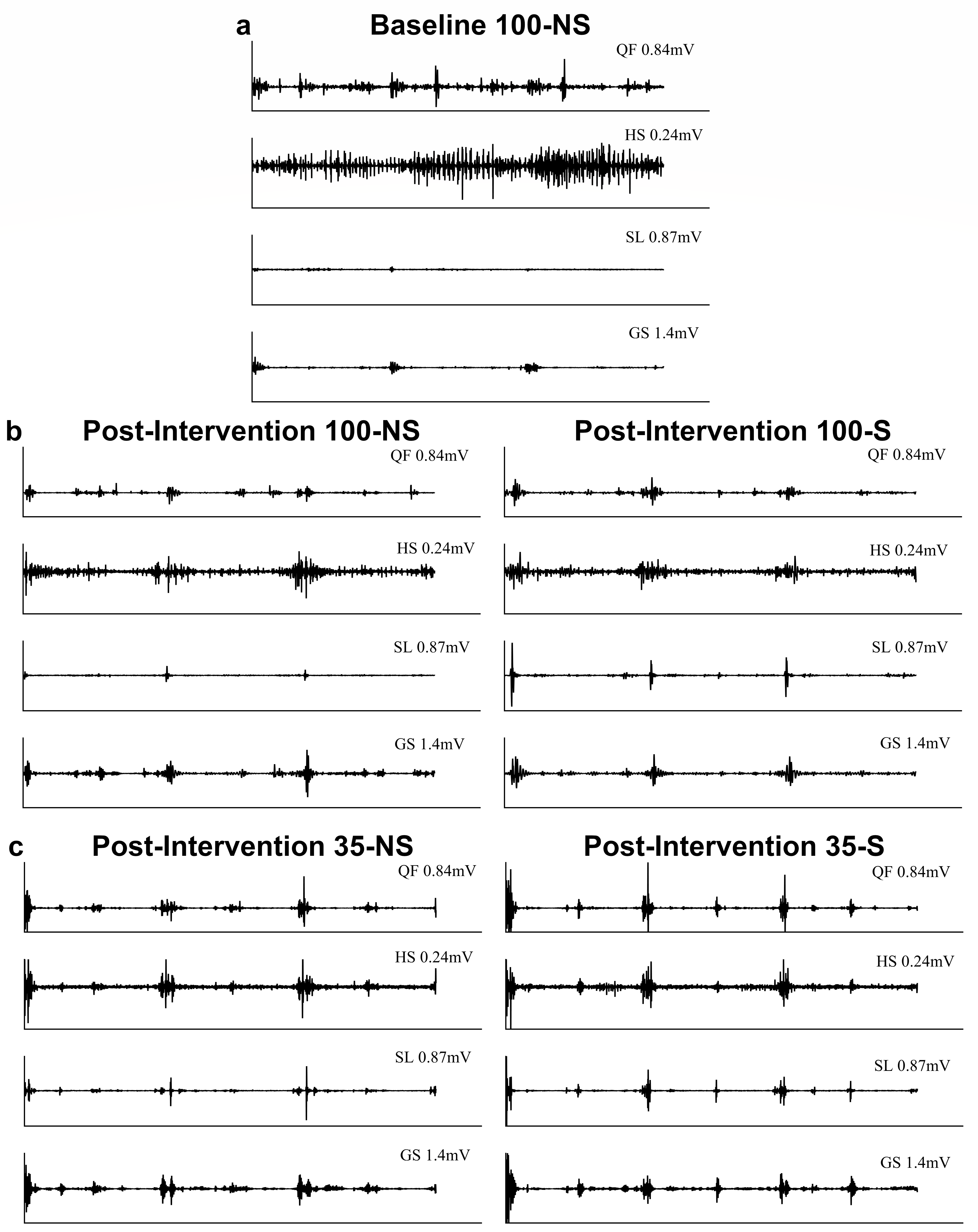

Supplement: Supplementary file 3 — Figure S3. Filtered EMG across three strides during week 0 (baseline) and week 13 (post‐intervention) of SCES enabled EAW. [file ACN3-7-259-s003.tiff]

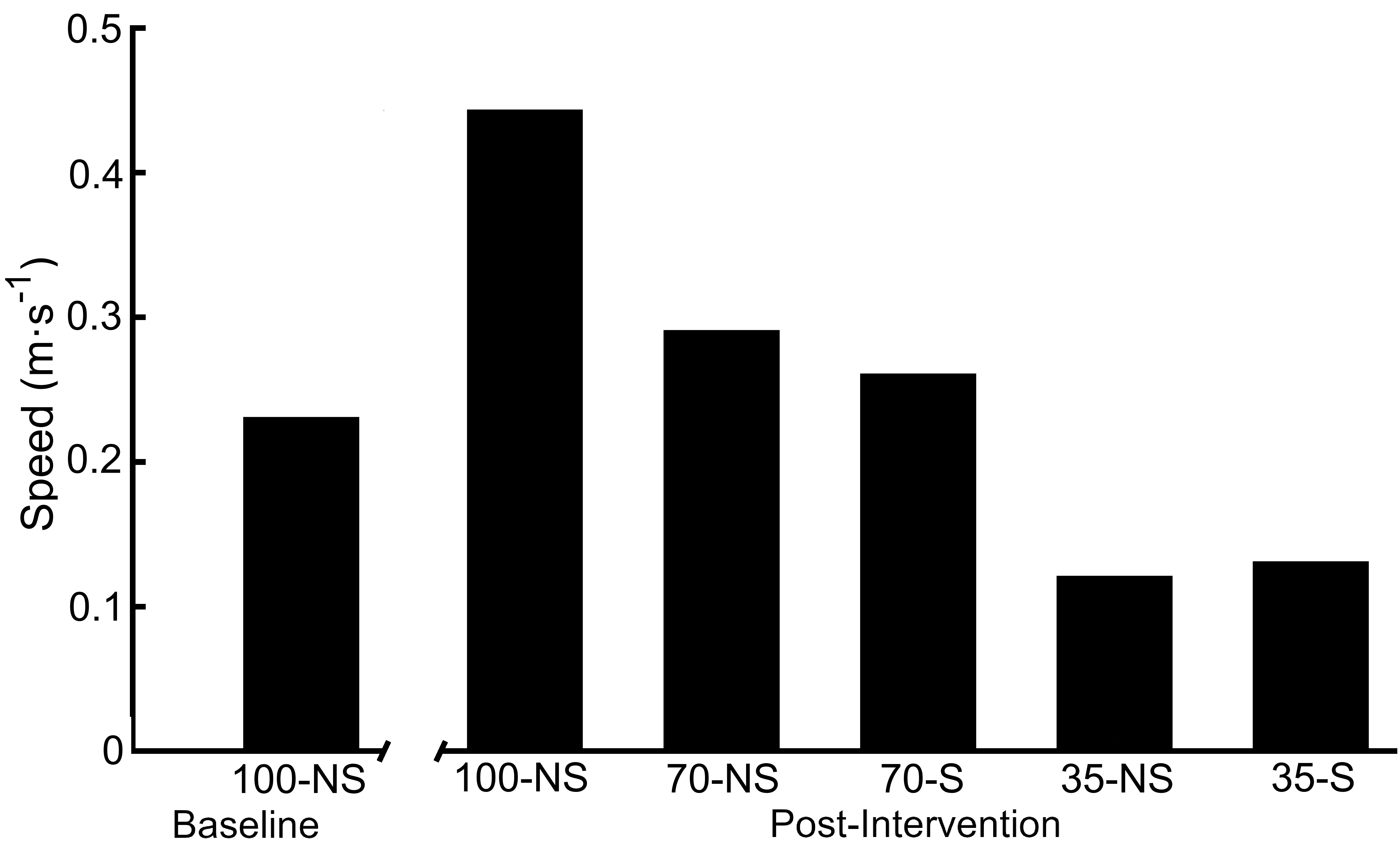

Supplement: Supplementary file 4 — Figure S4. Walking speed of SCES enabled EAW. Speed during 10 m walk test for various assistance levels at baseline and post‐intervention. [file ACN3-7-259-s004.tiff]
