# Supplementary material for: The feasibility of using exoskeletal‐assisted walking with epidural stimulation: a case report study
Source: Ann Clin Transl Neurol. 2020 Feb 5;7(2):259–65. doi: 10.1002/acn3.50983 (PMC7034511; doi:10.1002/acn3.50983)
Supplement: Supplementary file 5 — Table S1. Body composition assessment. [file ACN3-7-259-s005.docx]

|  | Arms | Legs | Trunk | Total |
| --- | --- | --- | --- | --- |
| %Fat, kg |  |  |  |  |
| Baseline | 21.1 | 30.1 | 38.4 | 32.3 |
| Post-intervention | 20.2 | 29.1 | 35.8 | 30.7 |
| Fat, kg |  |  |  |  |
| Baseline | 2.36 | 6.70 | 15.06 | 25.14 |
| Post-intervention | 2.17 | 6.49 | 13.8 | 23.40 |
| Lean mass, kg |  |  |  |  |
| Baseline | 8.41 | 14.82 | 23.29 | 50.03 |
| Post-intervention | 8.15 | 15.09 | 23.83 | 50.36 |
| BMC, kg |  |  |  |  |
| Baseline | .45 | .72 | .88 | 2.57 |
| Post-intervention | .44 | .72 | .88 | 2.56 |
| Total mass |  |  |  |  |
| Baseline | 11.2 | 22.3 | 39.2 | 77.7 |
| Post-intervention | 10.8 | 22.3 | 38.5 | 76.3 |

**Supplementary Table 1: Body composition assessment**
