# Supplementary material for: The feasibility of using exoskeletal‐assisted walking with epidural stimulation: a case report study
Source: Ann Clin Transl Neurol. 2020 Feb 5;7(2):259–65. doi: 10.1002/acn3.50983 (PMC7034511; doi:10.1002/acn3.50983)
Supplement: Supplementary file 6 [file ACN3-7-259-s006.docx]

**Supplementary**

**Ethical Approval.** This study was conducted in one subject who provided informed consent to EAW training study (ClinicalTrials.gov# NCT03410550). Ad hoc permissions were granted to allow the individual to activate his SCES during EAW, and to permit the research team to collect additional EMG measures. This case report was approved by the McGuire VA IRB.

**Description of Study Participant.** A 34-year-old male suffered a clinically complete SCI as a result of a snowboarding accident in 2014. The participant sustained a severe C7 complete SCI (American Spinal Injury Association Impairment Scale AIS A; supplemental Fig. 1) with sensory and motor zone of partial preservation at C8–T1. After 3 years and 9 months, the participant was implanted with an epidural stimulator (RestoreADVANCED, Medtronic, Fridley, MN; supplemental Fig. 2) using his own financial resources at a medical facility located outside U.S.A, which is not related to the study or to the research center. We could not determine the exact details of the surgical procedure, and this limitation may influence the repeatability and the outcomes of the current study.

The surgical procedure was followed by 40 days of inpatient rehabilitation with a focus on enhancing lower extremity motor control recovery. Daily rehabilitation was performed for 6 hours per day for 6 days per week. This involved adjusting the stimulation parameters, strengthening the paralyzed muscles and body weight supported treadmill training. Other activities involved sit to stand activity, balance exercise, and walking between parallel bars. We have adopted the established mapping pattern to activate his muscles during EAW

Eight months later, the participant enrolled in our 24 session (12 week) exoskeleton training study. At the time of admission, the participant weighed 77.5 kg, with height of 170 cm, and body mass index of 26.2 kg/m^2^. The participant had mild muscle spasms (1) as indicated by Penn Spasm Frequency Scale and spasticity ranged from 1 to 2 in the lower extremity muscles based on Modified Ashworth Scale testing. The participant also suffered from episodes of orthostatic hypotension and was instructed to be on Midodrine HCL (5 mg). Electrocardiography examination revealed that participant had a normal pattern. It should be noted that this participant had no previous experience with EAW. With SCES on, there was evidence of volitional control as witnessed by his ability to partially extend his leg during sitting in his wheelchair or sitting on the mat without back support.

**Experimental Design**: The original experimental design was constructed with the goal of assessing the effects of EAW on a number of cardiovascular and neuromodulatory outcomes. The enrollment of this participant presented a unique opportunity to investigate the combinatory effects of EAW + SCES.

In line with the original protocol, 24 sessions (12 weeks) of EAW training twice per week was carried out at the McGuire VA Medical Center. While in contrast to other studies providing up to 3–5 sessions per week for participants, we elected to use 2 sessions per week for up to 75 minutes per session. This aligns with recent SCI guidelines encouraging moderate to vigorous intensity training 2–3 times per week; additionally, this was thought to potentially increase subject adherence considering the physical and environmental barriers associated with SCI to participate in a longitudinal exercise trial.

Sessions 1–3 (weeks 1 and 2) were carried out to provide EAW training (no SCES) to the participant using 100% swing assistance. Between sessions 4 and 6 (week 2–week 3), various combinations of EAW swing assistance (100–95%) and SCES were tested to familiarize participant with EAW + SCES, as well as to explore the potential to reduce EAW swing assistance in future sessions. Beginning with session 7 (week 4), swing assistance provided by the exoskeleton was reduced based on the participant’s walking performance. The decision to drop the swing assistance was totally based on the subject’s ability to walk at least 45 minutes during a session. Dropping swing assistance resulted in an increase in intensity of EAW training as evidenced by increased RPE. This period was also used to train participant with EAW+SCES by encouraging the participant to turn on his epidural stimulator during portions of EAW sessions. The use of the SCES further increased the intensity of training as indicated by higher RPE during EAW+SCES. From session 17 to 24 (the last 4 weeks), the effect of EAW + SCES on locomotion was investigated by counting the number of EAW assisted as well as unassisted steps.

**SCES System.** In December 2017, our participant underwent implantation of a 16-electrode array SCES device (RestoreADVANCED, Medtronic, Fridley, MN) over spinal segments T12-S2 by performing laminectomy at the vertebral level of L1-L2. This intervention along with the subsequent muscle mapping was conducted outside of the United States by an independent surgical team unaffiliated with the authors or this research. The participant was able to control the output of his SCES device through an external programer unit (MyStim, Medtronic, Fridley, MN). All stimulation parameters except for the amplitude were defined and programed following the surgical implantation in an outside facility. The stimulation amplitude was adjusted based on subjective feedback from the participant.

**EAW Training.** Following study enrollment in July 2018, 24 sessions of EAW training were performed over a 12-week period. Training was performed twice a week and each session lasted for up to 75 minutes. During each session, the exoskeleton provided consistent body weight support while the swing assistance level was varied. This required that the participant shift his body weight to initiate stepping. When the level of swing assistance was dropped from 100%, the participant was provided with 2.5 seconds to initiate and complete stepping on his own. During this time, participant must move his leg from flexion (swing phase) into extension (stance phase) to be considered as an unassisted step. If the participant failed to achieve this in 2.5 seconds, the device will beep and passively move the leg into extension.

Participant was encouraged to initiate voluntary effort to move forward and maintain a predetermined trajectory. If participant was unable to provide enough voluntary effort, the device would then passively move the limb through the target range of motion. The participant was repeatedly encouraged to turn on his SCES and initiate unassisted steeping during EAW. While initial training was completed with the use of a walker, beginning with week 3 the participant switched to using bilateral Canadian crutches.

**Data Collection.** Throughout training, the exoskeleton device (EksoGT, v1.2) computed the total number of steps, walk time, and stand-up time at the end of each session. From session 17 to 24, the number of steps generated and completed by the participant’s volitional control (unassisted steps) during low assistance condition (< 100%) were counted by a research assistant based on auditory cues provided by exoskeleton.

At week 0 (baseline) and week 13 (post-intervention) EMG data were captured to evaluate muscle activity. Surface mounted EMG was recorded for the right LE over the QF, HS, SL, and GS muscle groups at 2000 Hz (Trigno Wireless, Delsys, Natick, MA).

**Data Processing.** The epidural stimulation artifacts were minimized from EMG data using band-pass filter (fourth order Butterworth, 20–450 Hz) using custom MATLAB scripts (MATLAB 2015a, The MathWorks Inc., Natick, MA). For baseline and post-intervention data, these filtered and continuous data were then visually segmented into individual strides by using the vertical component of an accelerometer embedded within the GS EMG sensor. This acceleration signal was low-pass filtered (fourth order Butterworth, 3 Hz) using custom MATLAB scripts. Each stride was further segmented into stance and swing phases based on this signal. EMG RMS envelopes were then calculated using a moving window of 150 samples for 10 strides. The timing and shape of these RMS envelopes was objectively compared using stride-to-stride cross-correlation analysis^22^. The cross-correlation measures the similarity between two curves as a scalar in the range of 0–1. Comparisons of these data provided objective measure of variability in the EMG signal between consecutive strides. These results for baseline and post-intervention are presented using R-values (R = 1 indicates exact agreement; two consecutive strides have same shape and timing). Peak filtered EMG values across the entire stride, each stance phase, and each swing phase were identified and averaged over the total number of strides completed during each assistance level.
